# Supplementary material for: COZOID: contact zone identifier for visual analysis of protein-protein interactions
Source: BMC Bioinformatics. 2018 Apr 6;19:125. doi: 10.1186/s12859-018-2113-6 (PMC5889581; doi:10.1186/s12859-018-2113-6)
Supplement: Supplementary file 2 — Software build. Executable binary file of the software tool. (ZIP 118,784 kb) [file 12859_2018_2113_MOESM2_ESM.zip › cozoid/platform/docs/testng-6.5.1-javadoc.zip!/org/testng/xml/package-summary.html]

org.testng.xml (TestNG)


---


|  |  |  |
| --- | --- | --- |
| **Overview** \| **Package** \| Class \| **Tree** \| **Deprecated** \| **Index** \| **Help** | |  |
| **PREV PACKAGE**   NEXT PACKAGE | **FRAMES**    **NO FRAMES**     **All Classes** |  |


---

## Package org.testng.xml

| **Class Summary** | |
| --- | --- |
| **XmlClass** | This class describes the tag  in testng.xml. |
| **XmlInclude** |  |
| **XmlMethodSelector** | This class describes the tag  in testng.xml. |
| **XmlPackage** | This class describes the tag  in testng.xml. |
| **XmlSuite** | This class describes the tag <suite> in testng.xml. |
| **XmlTest** | This class describes the tag <test> in testng.xml. |
| **XmlUtils** |  |

---


|  |  |  |
| --- | --- | --- |
| **Overview** \| **Package** \| Class \| **Tree** \| **Deprecated** \| **Index** \| **Help** | |  |
| **PREV PACKAGE**   NEXT PACKAGE | **FRAMES**    **NO FRAMES**     **All Classes** |  |


---
